# Effective vancomycin concentrations in children: a cross-sectional study

**DOI:** 10.31744/einstein_journal/2019AO4396

**Published:** 2019-02-06

**Authors:** Geisa Cristina da Silva Alves, Farah Maria Drumond Chequer, Cristina Sanches

**Affiliations:** 1 Universidade Federal de São João del-Rei, Divinópolis, MG, Brazil.; 2 Universidade de Itaúna, Itaúna, MG, Brazil.

**Keywords:** Microbial sensitivity tests, Pharmacokinetics, Pharmacologic actions, Vancomycin, Child, Testes de sensibilidade microbiana, Farmacocinética, Ações farmacológicas, Vancomicina, Criança

## Abstract

**Objective:**

Analyze the microbiological effectiveness, based on the pharmacokinetics/pharmacodynamics correlation of vancomycin in pediatric patients, and to propose dose adjustment.

**Methods:**

This is an observational, cross-sectional study, conducted in a pediatric hospital, over a 1-year period (2016 to 2017). Children of both sexes, aged 2 to 12 years, were included in the study; burn children, and children in renal replacement therapy were excluded. For the pharmacokinetic analysis, two samples of 2mL of whole blood were collected, respecting the 2-hour interval between each withdrawal.

**Results:**

Ten pediatric patients with median age of 5.5 years and interquartile range (IQR) of 3.2-9.0 years, median weight of 21kg (IQR: 15.5-24.0kg) and median height of 112.5cm (IQR: 95-133cm), were included. Only one child achieved trough concentrations between 10µg/mL and 15µg/mL.

**Conclusion:**

The empirical use of vancomycin in the children studied did not achieve the therapeutic pharmacokinetic/pharmacodynamic target for minimum inhibitory concentration of 1µg/mL.

## RESUMO

**Objetivo:**

Analisar a efetividade microbiológica considerando a correlação farmacocinética/farmacodinâmica de vancomicina em crianças e propor uma estimativa de ajuste na dose.

**Métodos:**

Trata-se de um estudo observacional, transversal, realizado em hospital pediátrico, no período de 1 ano (2016 a 2017). Foram incluídas crianças de 2 a 12 anos de ambos os sexos, tendo sido excluídas crianças queimadas ou submetidas à terapia renal substitutiva. Para análise farmacocinética, foram coletadas duas amostras de 2mL de sangue total, respeitando o intervalo de 2 horas entre cada coleta.

**Resultados:**

Foram incluídos dez pacientes pediátricos com idade de 5,5 anos (mediana) e intervalo interquartil (IQ) de 3,2-9,0 anos, peso de 21kg (mediana; IQ: 15,5-24,0kg) e altura de 112,5cm (mediana; IQ: 95-133cm). Apenas uma criança alcançou concentrações mínimas entre 10µg/mL e 15µg/mL.

**Conclusão:**

A utilização empírica de vancomicina na população de crianças não alcançou o alvo farmacocinético/farmacodinâmico terapêutico para concentração inibitória mínima de 1μg/mL.

## INTRODUCTION

In developing countries, the determination of regimen of antimicrobials in children is supported by empirical protocols, based on the etiological knowledge of infections, expert consensus, and linear reduction of adult doses.^(^ ^1^ ^)^

Patients who are critically ill, including pediatric patients, present age-related physiological changes, with consequent pharmacokinetic (PK) instability to antimicrobials, and may present changes in the apparent volume of distribution (Vd), plasma clearance, and reduced biological half-life.^(^ ^2^ ^,^ ^3^ ^)^

In addition, the therapeutic monitoring of antimicrobials is relevant and necessary to optimize pharmacotherapy and the selection of resistant bacteria, as well as to minimize the sub-therapeutic or toxic concentrations.^(^ ^2^ ^-^ ^6^ ^)^ Therefore, both safety and efficacy of antimicrobials related to the doses administered in children are questionable, since there are few studies conducted in this population on these therapeutic aspects.^(^ ^2^ ^,^ ^4^ ^,^ ^5^ ^,^ ^7^ ^)^

## OBJECTIVE

To analyze the microbiological effectiveness by means of the pharmacokinetic/pharmacodynamic correlation of vancomycin in pediatric patients and to propose a dose adjustment.

## METHODS

This study was approved by the Ethics Committee on Research Involving Human Beings, protocols CAAE: 44803815.7.0000.5545, and SIGED 3388/22712016, on March 17, 2016. The entire study was conducted in accordance with resolution 466/2012. Informed consent was obtained from every individual participants included in the study. The children included were invited to participate in the study and their legal guardians signed an Informed Consent, as well as an Agreement Term.

This is an observational, cross-sectional study conducted at a pediatric hospital in the Mid-Western region of the State of Minas Gerais, Brazil. Ten children on vancomycin, from March 2016 to March 2017, boys and girls, aged 2 to 12 years, were included in the study. Burn children and those undergoing renal replacement therapy were excluded.

The empirical dose of vancomycin used at the organization is 10 to 20mg/kg, according to guideline recommendations, to maintain adequate plasma concentrations.^(^ ^6^ ^)^

The variables of interest were obtained from the records of the children, comprising age, sex, weight, height and body mass index (BMI), presence of wounds, date of admission, hospitalization unit, first day of treatment, time of antimicrobial infusion, surgeries, central venous catheters, intra-arterial puncture, mechanical ventilation, serum creatinine, microbiological culture results and minimum inhibitory concentration (MIC). The cultures were obtained from biological specimens (blood, urine, catheter tip, lung, skin, eye and ear secretions). When not available in the medical records, the height and BMI of children were estimated from the anthropometric data available at the National Center for Health Statistics of the Centers for Disease Control and Prevention.^(^ ^8^ ^)^

The other variables of interest were obtained by equations involving creatinine clearance (CrCL), trough (Cmin), pharmacokinetic profile, Vd, elimination constant (Kel), antimicrobial half-life time (T_1/2_), and vancomycin clearance (CL).^(^ ^9^ ^)^

Renal clearance was estimated using the formula of Schwartz et al.,^(^ ^10^ ^)^ according to the equation 1:

CLcr = (HxK)/Scr___(Equation 1)

Where: H is height (cm); Scr is serum creatinine (mg/dL); K is constant related to age group and sex (K: 0.45 children <1 year; K: 0.55 children (1-12 years) and female adolescents; K: 0.70 male adolescents).

The Cmin and vancomycin Kel were determined from equation 2

$$\mathrm{Kel}\;=\;(\mathrm{lnC}1-\mathrm{lnC}2)/(T_{1}-T_{2})\_\_\_(\mathrm{Equation}\;2)$$

Where: C_1_ is the natural logarithm of the plasma concentration of the first sample; C_2_ is the natural logarithm of the plasma concentration of the second sample; T_1_ is the time of first blood sample; and T_2_ is the time of the second blood sample in relation to Cmin.

Equation 3 shows the determination of half-life of the antimicrobial T_1/2_:

$$T_{1/2}\;=\;0.693/\mathrm{Kel\_\_\_}(\mathrm{Equation}\;3)$$

Equation 4 shows the Vd:

$$\mathrm{Vd}\;=\;\mathrm{dose}*(e{\;}^{-\mathrm{KT}})/\mathrm{trough}*(1-e{\;}^{-\mathrm{KT}})\_\_\_(\mathrm{Equation}\;4)$$

Equation 5 shows the antimicrobial clearance:

CL = Vd*Kel___(Equation 5)

The area under the curve (AUC) of vancomycin was obtained using the software BestDose^™^, which is a clinical tool that uses non-parametric, multiple-model Bayesian adaptive control to calculate doses that achieve desired goals, such as serum drug concentration.^(^ ^11^ ^)^

The vancomycin efficacy prediction parameter considered for this study was the ratio of the AUC within 24 hours and the MIC above 400 (AUC^SS^_0-24_/MIC>400),^(^ ^6^ ^)^ and trough values between 10 to 15μg/mL.^(^ ^12^ ^,^ ^13^ ^)^

Respecting the interval of five biological half-lives of vancomycin, on the third day of treatment, two blood samples were collected at different times, with a minimum interval of 2 hours between them (2mL/collection in a sodium and EDTA Vacutainer^®^ tube) and duly identified. The samples were sent to the Laboratory of Toxicology of the *Universidade Federal de São João del Rei*, where they were centrifuged for 15 minutes at 3,500rpm. A total of 500μL of plasma was removed and stored in conical Eppendorf tubes. The samples were frozen at -80°C in a freezer (FORMA TM 88000 Series -86°C upright ultra-low temperature freezers) until the analysis was carried out, not exceeding 6 months.

The determination of vancomycin concentration in biological matrix (plasma) was conducted using a high performance liquid chromatograph (Agilent Technologies, model 1206), ChemStation for LC 3D systems software (Agilent Technologies^®^, USA), LiChroCART^®^ Purospher (MERCK^®^ C18), and 250mm per 4mm reversed phase column. The mobile phase was prepared daily from the mixture of ultrapure (UP) water and acetonitrile (9:1, v/v) added to 27g of potassium phosphate monobasic USP KH_2_PO_4_. The hydrogen potential (pH) of the solution was adjusted with hydrochloric acid to 3.0.

The injection volume for the analytical run was 40μL, and the effluent was monitored by an ultraviolet detector at 240nm. A 7-minute run time was required to detect the analyte and its internal standard (ceftriaxone), using the following concentration gradients: time 0, 100% mobile phase; from 2 to 5 minutes, 5% acetonitrile and 10% methanol were added to the mobile phase; from 5 to 7 minutes, returned to 100% mobile phase for stabilization.

The analyses were conducted at room temperature (20±1ºC). The daily calibration curve consisted of 8 points ranging from 2 to 100μg/mL. Internal controls were prepared at high quality control (HQC) concentrations (80.0μg/mL), medium quality control (MQC; 25.0μg/mL), and low-quality control (LQC; 3.0μg/mL. The plasma analyte was quantified based on the daily calibration curve, accepted by HQC, MQC and LQC.

Protein precipitation with acetonitrile was carried out for vancomycin purification in biological matrix, adding 200μL of plasma and 600μL of acetonitrile into a 1.5mL Eppendorf tube.

The samples were stirred in a vortex for 15 seconds, subjected to 8,000rpm, and refrigerated centrifugation at 4°C, for 30 minutes. After protein precipitation, the samples were concentrated by evaporation in nitrogen flow in a sample concentrator at 40ºC. The residue of the dry extract was dissolved in a solution with 100μL of UP water and acetonitrile in a ratio of (9:1, v/v), and the volume was transferred to microvials. The analytical method presented good linearity (r^2^=0.99), precision of 0.10 to 3.90, accuracy of 90.69 to 120.53, lower quantification limit of 2μg/mL, and limit of detection of 1.0μg/mL.

To estimate the ideal antimicrobial vancomycin dose, BestDose^™^ software and the equation suggested by Winter^(^ ^9^ ^)^ (Equation 6) were used.

$$\mathrm{desired}\;C^{\mathrm{ss}}\;=\;(\mathrm{desired}\;\mathrm{dose}\;\times \;\mathrm{current}\;C^{\mathrm{ss}})/\mathrm{current}\;\mathrm{dose}\;(\mathrm{Equation}\;6)$$

Where: C^ss^ is the concentration at steady state.

In order to estimate the vancomycin dose adjustment using the BestDose^™^ software, the defined dose interval was of 6 hours, with 60-minute infusion time, 7-day antimicrobial therapy duration, and MIC of 1μg/mL. The peak concentration in the second hour was 30μg/mL, and the trough concentration in the sixth hour was 10μg/mL.

Descriptive statistical analysis was conducted using medians, and dispersion, by an Excel spreasheet.

## RESULTS

Ten pediatric patients on vancomycin 10 to 20mg/kg/dose, median age of 5.5 years and interquartile range (IQR) of 3.2 to 9.0 years; median weight of 21kg and IQR of 15.5 to 24.0kg; and median height of 112.5cm and IQR of 95 to 133cm were included. Six (60%) participants were admitted to the intensive care unit, and 4 (40%) to the wards. The conditions leading to hospitalization were community acquired pneumonia (30%), cystic fibrosis (20%), bacterial pneumonia (10%), pulmonary sepsis (10%), bacterial meningitis (10%), craniectomy (10%), and septic shock (10%). The association between vancomycin and meropenem was found in four cases (40%). The individual characteristics of these children are shown in table 1.

**Table 1:** Anthropometric data and individual characteristics of the children

Children (10)	Sex (M/F)	Age (years)	Weight (kg)	Height (cm)	BMI (kg/m^2^)	SCr (mg/dL)	CrCl (mL/min)	Wounds (yes/no)
1	F	12	31	149	13.96	0.67	122.31	No
2	F	2	12.6	85	16.63	0.42	111.3	Yes
3	M	11	60	144	28.93	0.45	176	Yes
4	M	12	25.1	149	11.3	0.48	170.72	No
5	M	10	24.1	139	12.47	0.41	186.46	No
6	M	5	18	110	14.87	0.22	275	No
7	M	3	15	95	16.62	0.20	261.25	Yes
8	M	3	17	95	18.88	0.11	475	Yes
9	F	6	24	115	18.14	0.37	170.94	No
10	M	3	17	95	15.51	0.22	237.50	Yes
Patients	3/7	NAP	NAP	NAP	NAP	NAP	NAP	6 (60%)/4 (40%)
Median	-	5.5	21	112.5	16.06	0.41	181.23	-
Interquartile 25% - 75%	-	3.9-9.0	15.5-24.0	95-133	14.1-17.7	0.22-0.42	170.7-255.3	-
Vmin - Vmax	-	2-12.1	12.6/60	85/149	11.3/28.9	0.11/0.67	111.3/475	-

M: male; F: female; BMI: body mass index; SCr: serum creatinine; CrCl: creatinine clearance; NAP: not applicable; Vmin: minimum value; Vmax: maximum value.

The biological half-life of vancomycin in child number 3 is considerably increased (T_(1/2)ß_ 37.7 hours), as compared to other children; clearance, Kel and Vd were reduced. The child was obese and the dose used was based on recommendations for adults, as shown in table 2.

**Table 2:** Pharmacokinetic parameters of vancomycin in children

Children (n=10)	Trough	Pharmacokinetic parameters			
	
	Cmin _(μg/mL)_	^T^_(1/2)_β _(h)_	CL_T (mL/min)_	Kel _(h-1)_	Vd (L/kg)
1	2.76	4.17	2.61	0.23	0.67
2	14.3	9.48	0.29	0.10	0.17
3	22.6	37.7	0.01	0.02	0.04
4	3.85	4.31	2.79	0.22	0.76
5	4.82	4.37	2.23	0.22	0.60
6	2.69	4.37	4.06	0.22	1.10
7	5	4.37	1.43	0.22	0.39
8	2.68	2.98	3.64	0.32	0.67
9	3.66	1.93	2.17	0.49	0.26
10	4.4	7.78	1.24	0.12	0.60
Median	4.12	4.37	2.20	0.22	0.60
Interquartile 25-75%	2.98-4.95	4.20-6.93	1.29-2.65	0.14-0.27	0.29-0.66
Vmin/Vmax	2.68/22.6	1.93/37.7	0.019/4.06	0.02/0.49	0.04/1.10

Cmin: minimum trough concentration; ^t^(1/2)ß: biological half-life; CL: clearance total; Kel: elimination constant; Vd: volume of distribution.

For the PK and pharmacodynamic (PD) correlation of vancomycin. the individual values are shown in table 3. The MIC found in the results of cultures of biological material for vancomycin, issued by the pediatric hospital laboratory participating in this study, ranged from 0.5 to 2μg/mL. When the MIC of 0.5μg/mL was used, 100% of patients had effective vancomycin concentrations, whereas for MIC of 1μg/mL, only one child achieved the therapeutic target; for MIC of 2μg/mL, no child (0%) using vancomycin achieved the therapeutic target. Empirical treatment was initiated before having the culture results; however, three (30%) children had *Gram*-negative bacilli and were also on another antimicrobial to treat Gram-negative infection. The *Gram*-negative bacilli isolated were *Pseudomonas sp* in child 2, *Gram*-negative bacilli in child 3, and *Acinetobacter sp* in child 8. *Staphylococcus aureus* was isolated in four (40%) children, in blood cultures and pulmonary secretion. *Staphylococcus xylosus* was isolated in child 9. Out of ten children participating in the study, no bacteria were isolated in the culture of two (20%) children. The MIC of *Staphylococcus aureus* ranged from 0.5 and 2.0μg**/**mL.

**Table 3:** Pharmacodynamic profile of the study population for vancomycin

Children (n=10)	AUC^SS^_0-24_/MIC>400			

	AUC	0,5μg/mL	1μg/mL	2μg/mL
1	232	464	232	116
2	523	1046	523	261.5
3	308	616	308	154
4	322	644	322	161
5	385	770	385	192.5
6	362	724	362	181
7	250	500	250	125
8	200	400	200	100
9	382	764	382	191
10	295	590	295	147.5
Median	315	630	315	157.5
Interquartile 25-75%	261.2-377	522.5-754	261.2-377	130.6-188.5
Vmin/Vmax	200/523	400/1046	200/523	100/261.5

The MIC variation found in the results of biological material cultures were considered. AUC: area under the curve; Cmin: minimum trough concentration; Vmin: minimum value; Vmax: maximum value.

For each empirical dose regimen of vancomycin used in the children included in the study, dose adjustment was proposed using BestDose^™^ and guidelines for dose regimen suggested by Winter, according to table 4. Doses of 600mg and 841mg (median) were suggested to achieve the therapeutic target.

**Table 4:** Empirical daily dose regimen of vancomycin *versus* adjusted dose suggested by BestDose™ and Winter

Children (n=10)	Empirical dose	Frequency between doses (hour)	Infusion time	Dose per hour (mg)	BestDose™^*^ Dose per hour (mg)	Winter^**^ Dose per hour (mg)
1	10mg/kg	6/6	1 hour	310	600	1,122
2	10mg/kg	6/6	1 hour	126	100	87.7
3	8.4mg/kg	6/6	1 hour	500	950	381
4	15mg/kg	6/6	1 hour	376	800	977
5	15mg/kg	6/6	1 hour	362	600	749
6	15.3mg/kg	6/6	1 hour	275	470	1,020
7	10mg/kg	6/6	1 hour	150	300	300
8	15mg/kg	6/6	1 hour	250	800	932
9	20mg/kg	6/6	1 hour	480	730	1,309
10	10mg/kg	6/6	1 hour	150	250	339
Median	12.5	-	1 hour	292.50	600	841
Interquartile 25-75%	10-15	-	1 hour	175-372.8	342-782.50	349-966
Vmin/Vmax	8.4/20	-	1/1	126/500	100/950	87/1,309

* Considering BestDose^™^: frequency of 6 hours, MIC of 2mg/L, time of therapy 7 days, peak concentration of 30μg/mL in the second hour and trough of 10μg/mL in the sixth hour. ** Considering the formula according to Winter et al.:^(^ ^9^ ^)^ desired concentration (10μg/mL) = (desired dose) x (current C^ss^ current)/current dose.

## DISCUSSION

Uncertainty regarding the use of efficacy parameters and therapeutic levels in children is still a reality. Children have not fully benefited from treatment and understanding of medication toxicity in the clinical progression of infection.^(^ ^14^ ^,^ ^15^ ^)^ Recommendations for using vancomycin have undergone major changes over the years; yet, further studies are required to demonstrate evidence of efficacy of antimicrobial therapeutic monitoring in children.^(^ ^14^ ^,^ ^16^ ^)^ The present study showed the empirical doses of vancomycin administered in children were unable to guarantee microbiological efficacy for both the Cmin (trough) and AUC/MIC parameters for MICs of 1 and 2mg/L. Although the empirical doses used follow the recommendations of guidelines, according to Ye et al.,^(^ ^17^ ^)^ the therapeutic monitoring of vancomycin is associated with higher clinical efficacy rates in patients with *Gram*-positive infections. A recent review by Alves et al.,^(^ ^18^ ^)^ stated that in addition to dose individualization, the use of initial doses of vancomycin >60mg/kg/day is also necessary to obtain effective concentrations of the drug.^(^ ^2^ ^)^

These findings reinforce the difficulty of establishing an empirical dose for children, and it is necessary to develop models that take into account the many characteristics of this population. In addition, these findings also indicate the need for therapeutic monitoring of vancomycin, and should be a matter of concern regarding the use of antimicrobials in pediatric units, since the doses should be adjusted according to the profile of each child, to obtain an adequate therapeutic response.^(^ ^2^ ^)^

Regarding the microbiological efficacy profile of vancomycin, there are two parameters available and currently used: the therapeutic ranges for trough and the PK/PD correlation. According to Rybak et al.,^(^ ^6^ ^)^ trough concentrations between 10 and 20μg/mL are determined to reflect an AUC/MIC >400 in adults. It is therefore preferable, when possible, to measure the PK/PD. In our study this knowledge is extremely important for clinical practice, and presents a very relevant implication. In other words, it is observed that in patients presenting baseline concentrations within and above the therapeutic range established by the formula suggested by Winter et al.,^(^ ^9^ ^)^ which takes into account the desired trough, would need of the dose reduction. On the other hand, when analyzing the PK/PD correlation for these same subjects, the therapeutic target was not achieved for MIC of 1 and 2μg/mL, and the dose administered had to be increased, according to the estimate of dose adjustment provided by the software BestDose^™^.

Another important finding in our study when we considered the serum concentration of vancomycin in the trough was that an obese child (number 3) presented serum trough levels greater than 20μg/mL, using a dose of 8.4mg/kg; however, they did not achieve serum levels to maintain AUC^SS^_0-24_/MIC >400. Zhao et al.,^(^ ^19^ ^)^ reported weight and renal function have a significant impact on the PK of vancomycin in children, because the clearance of both vancomycin and creatinine is increased in direct proportion to body weight. Population models that take into account the age, weight and clinical conditions of the pediatric population must be developed and made available for clinical practice.

Although the BestDose^™^ software is based on the American pediatric population, it was developed to be used specifically in children; dose estimation is suggested according to the individual profile of the formula suggested by Winter et al.^(^ ^9^ ^)^ Using BestDose^™^ software, the most appropriate dose can still be adapted so that vancomycin concentrations remain within the trough and AUC parameters. Therefore, due to the lack of an instrument for dose adjustment for the Brazilian population, or even the lack of therapeutic drug monitoring services in health organizations in Brazil, BestDose^™^ is a reliable tool for dose adjustment in children. There are no population models of vancomycin in the pediatric population in Brazil.^(^ ^2^ ^,^ ^20^ ^,^ ^21^ ^)^

Much has been argued in relation to toxicity, mainly with the use of vancomycin. In our study, some endpoints, such as nephrotoxicity, were not evaluated. A study by Cole et al.,^(^ ^16^ ^)^ found that the scientific evidence on vancomycin nephrotoxicity is associated with the concurrent use of other nephrotoxic drugs, especially in critically ill children on polypharmacy. For Ye et al.,^(^ ^17^ ^)^ therapeutic monitoring of vancomycin is associated with lower nephrotoxicity rates.

This study has the limitation of a small sample of children who met the inclusion criteria in the period. In addition, during this period, data on adverse events related to the use of vancomycin were not recorded.

## CONCLUSION

The empirical use of vancomycin in the studied population did not achieve the therapeutic pharmacokinetic and pharmacodynamics target for minimum inhibitory concentration in most cases, and dose adjustment was necessary. These findings highlight the importance of implementing therapeutic monitoring of vancomycin in severely ill children, with potential pharmacokinetic changes, aiming to reach the therapeutic target and improve clinical response. This study demonstrated the urgent need of a dose adjustment instrument for the Brazilian pediatric population profile.
